# Single or Combined Dietary Supply of *Psidium guajava* and *Phyllanthus amarus* Extracts Differentially Modulate Immune Responses and Liver Proteome in Striped Catfish (*Pangasianodon hyphophthalmus*)

**DOI:** 10.3389/fimmu.2020.00797

**Published:** 2020-05-05

**Authors:** Truong Quynh Nhu, Bui Thi Bich Hang, Valérie Cornet, Mathilde Oger, Le Thi Bach, Nguyen Le Anh Dao, Do Thi Thanh Huong, Joëlle Quetin-Leclercq, Marie-Louise Scippo, Nguyen Thanh Phuong, Patrick Kestemont

**Affiliations:** ^1^Research Unit in Environmental and Evolutionary Biology (URBE), Institute of Life, Earth and Environment, University of Namur, Namur, Belgium; ^2^College of Aquaculture and Fisheries, Cantho University, Cantho City, Vietnam; ^3^College of Natural Sciences, Cantho University, Cantho City, Vietnam; ^4^Pharmacognosy Research Group, Louvain Drug Research Institute, Université Catholique de Louvain, Brussels, Belgium; ^5^Laboratory of Food Analysis, Department of Food Sciences, Faculty of Veterinary Medicine, Fundamental and Applied Research for Animals & Health, Veterinary Public Health, University of Liège, Liège, Belgium

**Keywords:** liver proteome profile, humoral immune response, *Edwardsiella ictaluri*, plant extract-based diets, *Phyllanthus amarus* Schum et Thonn, *Psidium guajava* L, skin mucosal immune response, striped catfish

## Abstract

Guava *Psidium guajava* L (Pg) and bhumi amla *Phyllanthus amarus* Schum. et Thonn (Pa) are well-known plants in traditional medicine. However, the capacity of these plants for improving the immune system of aquatic species has received less attention so far. This study aimed to investigate the effects of single supply or mixture of Pg and Pa extracts on immune responses, disease resistance and liver proteome profiles in striped catfish *Pangasianodon hypophthalmus*. Fish were fed diets including basal diet 0% or one of three doses of each plant extract, either alone or in mixture, 0.08, 0.2, or 0.5% Pg, Pa or mixture (Pg:Pa, v/v) for 6 weeks. The immune parameters (respiratory burst activity (RBA); nitric oxide synthase (NOS), total immunoglobulin, lysozyme, and complement activities) were examined at W3, W6 post-feeding, and after challenge test. The growth parameters and the challenge test with *Edwardsiella ictaluri* were done at W6. The liver proteome profiles were analyzed in W6 at 0.08 and 0.5% of each extract. The results showed that extract-based diets significantly improved growth parameters in the Pg0.2 group compared to control. The cellular immune responses in spleen and the humoral immune responses in plasma were significantly improved in a dose and time-dependent manner. Diets supplemented with single Pg and Pa extracts, and to lesser extent to combined extracts, could significantly decrease the mortality of striped catfish following bacterial infection compared to control. The proteomic results indicated that some pathways related to immune responses, antioxidant and lipid metabolism were enriched in liver at W6. Several proteins (i.e., CD8B, HSP90AA1, HSP90AB1, PDIA3, CASP8, TUBA1C, CCKAR, GNAS, GRIN2D, PLCG1, PRKCA, SLC25A5, VDAC2, ACTN4, GNAI2, LCK, CARD9, NLRP12, and NLRP3) were synergistically upregulated in mixture of Pg and Pa-based diets compared to control and single dietary treatments. Taken together, the results revealed that single Pg and Pa extracts at 0.2 and 0.5% and their mixture at 0.08 and 0.5% have the potential to modulate the immune mechanisms and disease resistance of striped catfish. Moreover, the combination of Pg and Pa in diets suggested positive synergistic effects liver proteome profile related to immune system processes.

## Introduction

Infectious pathogens (e.g., viruses, bacteria, parasites, and fungi) causing high mortality rates are a major problem leading to tremendous economic losses in aquaculture production worldwide (1, 2). Many approaches have been successfully applied to overcome the obstacles of infectious diseases. In recent years, increasing numbers of scientific reports are interested in strategies for green growth in order to promote a more sustainable aquaculture sector. Environmentally friendly prophylactic measures based on dietary supplementation of natural plant products have been widely developed to enhance the immune system, reduce mortality, and improve growth performance in cultivated aquatic animals (3). Among plants with potential medical interest, guava *Psidium guajava* (Pg) and bhumi amla *Phyllantus amarus* (Pa) are known for their pharmacological activities including anti-bacterial, anti-stress and immune response functions. Active ingredients of Pg and Pa include alkaloids, steroids, phenols, tannins, terpenoids, saponins, glycosides, flavonoids, and many other compounds such as polysaccharides (4–7). Earlier studies reported that dietary supplementation with Pg extracts significantly improved the growth performance, antioxidant, and immune parameters in rohu *Labeo rohita* (8, 9), Mozambique tilapia *Oreochromis mossambicus* (10) and common carp *Cyprinus carpio* (11). To the best of our knowledge, there are a limited number of publications so far on the effects of Pa extract-enriched diets on immune responses of aquatic species. Only the study by Sundaram et al. (12) has demonstrated that acetone and petroleum ether extracts of Pa could protect the freshwater crab *Paratelphusa hydrodomous* against white spot syndrome virus. Based on the results of the *in vitro* evaluation the capacity of 20 plant extracts to enhance the immunity of striped catfish *Pangasianodon hypophthalmus* (13), we previously found that five ethanol plant extracts including Pa, Pg, sensitive plant *Mimosa pudica* L., neem *Azadirachta indica* A. Juss and asthma plant *Euphorbia hirta* L. were potentially interesting for modulating blood parameters, immune responses and providing better protection to striped catfish against infection with the pathogenic bacteria *Edwardsiella ictaluri* after 8 weeks of feeding (14).

The teleost immune system differs in several ways from mammals, the innate immune system being usually considered as more efficient than the one of mammals while the acquired immunity might be weaker (15). For example, the teleost humoral immunity including complement or cytokines involved in activation and key signaling pathways (e.g., toll-like receptors, lectins, peptidoglycan recognition proteins, and cytokine receptors) displays higher diversity compared to mammals (15). On the other hand, fish acquired immune system lacks of bone marrow, lymph nodes, and germinal centers (16).

The innate immune response is a first line defense mechanism to eliminate invading pathogens (e.g., bacteria, viruses, fungi, protozoans, and parasites). Physical properties of the innate and adaptive immune response including lysozymes, complement, lectins, transferrin, agglutinins, lysine, and immunoglobulins act as biological defense molecules in fish. In aquaculture, the elevation of immune parameters is a useful tool to assess whether immunostimulants affect the fish immune responses. Additionally, proteomic approaches have been recently developed as a powerful tool to interpret specific influences of exercise or diet on the metabolic process of the immune system (17). Among the organs, the liver represents an important organ for metabolism, nutrient storage, and immune responses by producing cytokines, chemokines and complement components (18). Mendez et al. (2017) demonstrated the capacity of high-fat and sucrose-based diets to enhance fatty acid beta-oxidation, insulin signaling, ameliorate endoplasmic reticulum stress and protein oxidation pathways in rat liver (19). Under low-temperature stress, D-arginine and D-ornithine metabolism, MAPK signaling, and Wnt signaling pathways were significantly enhanced in obscure pufferfish *Takifugu fasciatus* liver (20). Causey et al. (2018) reported that the immune function and liver metabolism in rainbow trout *Oncorhynchus mykiss* promoted rewiring in host defense responses during infection with *Aeromonas salmonicida* (21). Semi-synthetic diets enriched with different sources of proteins including soybean, fish, chicken, pork, or beef altered the metabolism in rat liver, resulting in mediated antioxidant and anti-inflammatory responses (22). With regard to diets supplemented with plant extracts, no publications so far have addressed the link between protein expression levels and plant extracts in fish liver. In the present study, we evaluated the effects of different dietary doses of Pg and Pa (alone or in combination) on growth performance, immune parameters and disease resistance against *E. ictaluri* in striped catfish. In addition, a gel-free proteomic analysis was carried out using the fish liver to better understand the metabolic pathways involved when the selected plant extracts, alone or in combination, were added to the diet of striped catfish.

## Materials and Methods

### Diet Preparation

The fresh part of Pg (leaves) and Pa (leaves, twigs) were extracted by using 96% ethanol solvent as previously described (14). The experimental diets were prepared using the extract ingredients to contain 0, 0.08, 0.2, and 0.5% of each Pg or Pa extract and their mixture (v: v) at similar concentrations (Table 1). The basal and experimental diets were prepared following the previous study (14) and pellets of 2 mm were stored at −20°C until use.

**TABLE 1:** Composition of experimental diets.

**Ingredients (100 g of feed)**	**Control diet**	**Experimental diets**								
		**Pg0.08**	**Pg0.2**	**Pg0.5**	**Pa0.08**	**Pa0.2**	**Pa0.5**	**Mix0.08**	**Mix0.2**	**Mix0.5**
^a^Soybean meal (g)	24	24	24	24	24	24	24	24	24	24
^b^Rice bran (g)	29.5	29.5	29.5	29.5	29.5	29.5	29.5	29.5	29.5	29.5
^c^Casava (g)	17.96	17.88	17.76	17.46	17.88	17.76	17.46	17.80	17.56	16.96
^d^Fishmeal (g)	24	24	24	24	24	24	24	24	24	24
^e^Fish oil (g)	1	1	1	1	1	1	1	1	1	1
^f^Premix* (g)	3	3	3	3	3	3	3	3	3	3
Phytase	0.02	0.02	0.02	0.02	0.02	0.02	0.02	0.02	0.02	0.02
^g^Gelatin (g)	0.5	0.5	0.5	0.5	0.5	0.5	0.5	0.5	0.5	0.5
^h^Butylated hydroxytoluene (BHT)	0.02	0.02	0.02	0.02	0.02	0.02	0.02	0.02	0.02	0.02
**Plant extracts (g)**										
Pg	–	0.08	0.2	0.5	–	–	–	–	–	–
Pa	–	–	–	–	0.08	0.2	0.5	–	–	–
Mixture	–	–	–	–	–	–	–	0.08	0.2	0.5

*Pa, *Phyllanthus amarus*, Pg, *Psidium guajava*, Mix, Mixture. ^*a*^Wilpromil R Soy Protein Concentrate, Yihai (Fangchenggang) Soybeans Industries, Wilmar Group, Fangchenggang, China. ^*b*^Cai Lan Oils & Fats Industries Company, Can Tho Branch, Can Tho City, Vietnam. ^*c*^Hong Ha Company, Can Tho City, Vietnam. ^*d*^Minh Tam, Can Tho, Vietnam. ^*e*^Vegetable oil (Simply, Vietnam) and squid oil (Vemedim, Vietnam) at a ratio of 1:1. ^*f*^The vitamin/mineral premix (Unit/kg) from Vemedim, Can Tho, Vietnam: vitamin A, 6,000 IU; vitamin D3, 5600 IU; vitamin E, 160 IU; vitamin B1, 10 mg; vitamin B6, 20 mg; vitamin B12, 0.03 mg; vitamin K, 0.3 mg; riboflavin, 60 mg; vitamin C, 300 mg; pantothenic acid, 60 mg; folic acid, 8 mg; nicotinic acid, 184 mg; biotin, 0.3 mg; iron, 50 mg; copper, 10 mg; iodine, 9 mg; zinc, 34 mg; selenium, 0.4 mg; manganese, 30 mg. ^*g*^Xilong Chemical Industry Incorporated, China. ^*h*^Honshu Chemical Industry Company, Japan.*

### Fish

Farm-raised striped catfish juveniles (15–20 g) obtained from a local fish farm in Vinh Long province of Vietnam were transported to the laboratory in plastic bags filled with oxygenated water. The fish were acclimatized to laboratory conditions for 15 days and then stocked into composite tanks (250 L) in a flow through a freshwater supply system, and fed twice a day with the formulated diet at a rate of 2% of their body weight/day.

### Bacteria Preparation

*E. ictaluri* strain - Ed1 were cultured on tryptic soy agar plates (TSA, Merck) for 48 h at 28°C following the previous study by Hang et al. (23). Then, a single colony was collected and harvested into tryptic soy broth (TSB, Merck). This suspension was shaken overnight, at 180 rpm and 28°C. Then, bacteria were centrifuged at 5,000 rpm at 4°C for 5 min and washed 3 times with 0.85% NaCl solution. The mean colony count used the optical density method (24) and OD was adjusted to a value of 0.1 by spectrophotometer (Thermospectronic, United States) at 590 nm. Then, this suspension was diluted 1,000 times with NaCl solution before injection into the fish.

### Experimental Design

For the feeding trial of extract-based diets, fish were randomly distributed into ten distinct treatments, each treatment in triplicate. Fish were fed the experimental diets described above for 6 weeks, at 2% of body weight and three times (8 am, 12 am, and 5 pm) daily. The tank capacity was 250 L, each tank contained 45 fish. The photoperiod was fixed at 12 h light: 12 h dark. Water temperature, dissolved oxygen, and pH were monitored daily and maintained at 30 ± 2°C, 5.7 ± 0.01 mg L^–1^, 7.5 ± 0.02, respectively, throughout the experimental period. The daily feed supplied was recorded, and the uneaten feed was collected 1 h after feeding by syphoning, followed by drying, weighing, and finally subtracted from the total amount of supplied feed to calculate the actual feed intake.

After 6 weeks of feeding, all groups (45 fish/group) fed plant extract-based diets were injected intraperitoneally with 0.1 mL LD50 (2 × 10^6^ colony-forming unit/mL) of *E. ictaluri* suspension. At the same time, the control groups were divided into two small groups, the first one was the control injected with 0.1 mL of 0.85% NaCl solution and the second one challenged with 0.1 mL LD50 of *E. ictaluri*. All groups were maintained in triplicate, 15 fish per tank. Cumulative mortality was recorded daily for 14 days after the challenge test. In order to be sure that the mortalities were due to the bacterial infection, *E. ictaluri* was re-isolated and identified by PCR confirmation as described in (23).

### Sample Collection

The skin mucus samples were collected at W3 and W6 of the feeding trial, and 3 days post injection (3 dpi) according to Ross et al. (25) with slight modifications. Briefly, 3 fish per tank were randomly collected and anesthetized using 0.1 ppm of M222 (Sigma-Aldrich, United States). Fish surfaces were individually washed with distilled water and then transferred into polyethylene bags containing 1 mL of PBS 1X. After 2 min with gentle shaking, mucus was collected, transferred to 2.0 mL sterile Eppendorf tubes and centrifuged (1500 × *g* for 10 min at 4°C). The supernatant was stored at –80°C for further analysis. Similarly, blood samples were obtained from the caudal vein of individual fish (9 fish per treatment, 3 fish per tank) and centrifuged at 4,000 rpm for 10 min. The plasma supernatant was collected into new Eppendorf tubes and kept at –80°C until analysis. At the end of the feeding trial, all experimental fish were weighed for growth performance calculations.

### Growth Performance

All fish were deprived of food 24 h before weighing and sampling, and the following parameters were measured at the end of the feeding trial (6 weeks):

Weight gain (WG) = 100 × (W2 – W1)/W1
Specific growth rate (SGR) = 100 × (ln W2 – ln W1)/T
Feed conversion ratio (FCR) = feed intake (g)/weight gain (g)
Where W1 is the initial weight (gram), W2 is the final weight (gram) and T is the number of days in the feeding period.

## Cellular Immune Variables

### Spleen Respiratory Burst Assay

Respiratory burst was adapted from Rock et al. (26). Spleens were weighed and then mashed in L-15 medium (Saint Louis, MO, United States) through a 100 μM nylon mesh. Cell suspensions were washed and centrifuged (1,000 × *g*, 5 min, 28°C) twice in L-15 medium. The culture media were then replaced by the corresponding fresh culture media containing 2 mg ml^–1^ nitroblue tetrazolium (NBT). Cells were incubated for 1 h at 28°C in a light protected environment. After 1 h, the cells were washed twice in PBS and the reaction was stopped by adding 200 μL of methanol. The cells were rinsed by centrifugation (1,000 × *g*, 10 min, 4°C) and finally air dried for 10 min. Resulting formazan was dissolved in 240 μL of KOH 2M and 280 μL of N-dimethylformamide. The absorbance of the final supernatant was measured at 550 nm. A standard curve was produced using serial dilutions of NBT directly dissolved in KOH 2M and N-dimethylformamide. Samples and negative control without cells were performed in duplicate. Activity was reported on protein concentration in spleen measured by Bradford assay.

### Spleen Nitric Oxide Species Assay (NOS)

Production of NOS was measured by the Griess reaction. First, 100 μL of cell suspensions collected from the spleen were incubated with 5 μL of *E. ictaluri* suspension (OD 2) resuspended in corresponding culture media for 1 h at 28°C. Then, 100 μL of the Griess reactant was added and solutions were incubated for 15 min. The absorbance was measured at 540 nm. The activity was reported for protein concentration in spleen measured by Bradford assay.

## Humoral Immune Variables

### Lysozyme Assay

The lysozyme assay protocol was adapted from Ellis (27) and Milla et al. (28). In 96–well microplates, the lysozyme activity assay was initiated by mixing 10 μL of plasma or 20 μL of skin mucus with 130 μL of lyophilized *Micrococcus lysodeikticus* (Sigma) suspension in phosphate buffer, pH 6.2 (0.6 mg mL^–1^ for plasma and 0.3 mg mL^–1^ for skin mucus). The difference in absorbance at 450 nm was monitored between 0 and 30 min for plasma (0 and 15 min for the skin) and used to calculate lysozyme activity in units. One unit represents the amount of lysozyme that caused a 0.001 decrease in absorbance.

### Complement Assay

The plasma alternative complement pathway was assayed using rabbit red blood cells (RRBC, Biomerieux, Craponne, France) as targets following Sunyer and Tort (29) and adapted by Milla et al. (28). Briefly, 10 μL of RRBC suspension (3%) diluted in veronal buffer (Biomerieux) was mixed with serial dilutions of plasma (60 μL total volume). After incubation for 100 min at 28°C, the samples were centrifuged at 2,000 × *g* for 10 min at room temperature. The spontaneous hemolysis was obtained by adding 60 μL of veronal buffer to 10 μL of RRBC. The total lysis was obtained by adding 60 μL of distilled water to RRBC. The absorbance was measured at 405 nm.

### Total Ig Assay

The total immunoglobulin concentration was measured using the method of Siwicki and Anderson (30), modified by Milla et al. (28). Briefly, immunoglobulins were precipitated with 10,000 kDa polyethylene glycol (PEG, Sigma). Plasma or skin mucus samples were mixed with 12% PEG solution (v:v) for 2 h at room temperature under constant shaking. After centrifugation at 1,000 × *g* for 10 min, the supernatant was collected and assayed for its protein concentration. The total immunoglobulin concentration was calculated by subtracting this value from the total protein concentration in the plasma before precipitation with PEG.

### Statistical Analyses

All statistical analyses were performed using SPSS version 20. Results are presented as means ± SEM (standard error of the mean). The normality of the data and the homogeneity of variance between groups were tested using Shapiro-Wilks and Levene tests. One-way analyses of variance (ANOVA) and Duncan’s multiple range test at a confidence level of 95% (*p* < 0.05) were used to determine significant differences between immunological variables in fish from the different plant extract treatments and control treatment.

## Liver Quantitative Proteomic Analysis

### Protein Extraction and Digestion

Proteomic analysis was performed on liver samples at low (0.08%) and high (0.5%) doses of each kind of plant extract in W6. Three fish from the same tank were pooled and tanks were considered as independent biological replicates. Briefly, samples were ground to powder and the powder was dissolved in lysis buffer 8 M urea and 40 mM Tris–HCL or triethylammonium bicarbonate (TEAB; pH 8.5) supplemented with 1 mM phenylmethylsulfonyl fluoride and 2 mM ethylenediaminetetraacetic acid to a final concentration of 10 mM, and the suspension was sonicated at 200 W for 1 min prior to centrifugation at 25,000 × *g* for 20 min at 4°C. Proteins were reduced using 5 mM DTT (dithiothreitol) and alkylated using 15 mM iodoacetamide. Proteolysis was performed with 0.5 μg of trypsin and allowed to continue overnight at 37°C. Each sample was dried under vacuum using a Savant Speedvac Concentrator.

## LC-IMS (Ion Mobility Separation)-QTOF-MS Analysis (HDMSE)

### Peptide Separation Using nanoUPLC

Before peptide separation, the samples were dissolved in 20 μL of 0.1% (v/v) formic acid and 2% (v/v) acetonitrile (ACN). The peptide mixture was separated by reverse phase chromatography on a NanoACQUITY UPLC MClass system (Waters, MA, United States) working with MassLynx V4.1 (Waters, MA, United States) software. 200 ng of digested proteins were injected on a trap C18, 100 Å 5 μm, 180 μm × 20 mm column (Waters, MA, United States) and desalted using isocratic conditions with a flow rate of 15 μL/min using a 99% formic acid and 1% (v/v) ACN buffer for 3 min. The peptide mixture was subjected to reverse phase chromatography on a C18, 100 Å 1.8 μm, 75 μm × 150 mm column (Waters, MA, United States) PepMap for 120 min at 35°C and a flow rate of 300 nL/min using a two part linear gradient from 1% (v/v) ACN, 0.1% formic acid to 35% (v/v) ACN, 0.1% formic acid, and from 35% (v/v) ACN, 0.1% formic acid to 85% (v/v) ACN, 0.1% formic acid. The column was re-equilibrated to initial conditions after washing for 10 min at 85% (v/v) ACN, 0.1% formic acid at a flow rate of 300 nL/min. For online LC-MS analysis, the nanoUPLC was coupled to the mass spectrometer through a nano-electrospray ionization (nanoESI) source emitter.

IMS-HDMS^E^ (ion mobility separation-high definition enhanced) analyses were performed on a SYNAPT G2-Si high definition mass spectrometer (Waters, MA, United States) equipped with a NanoLockSpray dual electrospray ion source (Waters, MA, United States). Precut fused silica PicoTip^R^ emitters for nanoelectrospray, outer diameter: 360 μm; inner diameter: 20 μm; 10 μm tip; 2.5 inches length (Waters) were used for samples and precut fused silica TicoTip^R^ emitters for nanoelectrospray, outer diameters: 360 μm; inner diameter: 20 μm; 2.5 inches length (Waters, MA, United States) were used for the lock mass solution. The eluent was sprayed at a spray voltage of 2.4 kV with a sampling cone voltage of 25 V and a source offset of 30 V. The source temperature was set to 80°C. The HDMS^E^ method in resolution mode was used to collect data from 15 min after injection to 106 min. This method acquires MS^E^ in positive and resolution mode over the m/z range from 50 to 2,000 with a scan time of 1 sec. with a collision energy ramp starting from ion mobility bin 20 (20 eV) to 110 (45 eV). The collision energy in the transfer cell for low-energy MS mode was set to 4 eV. For the post-acquisition lock mass correction of the data in the MS method, the doubly charged monoisotopic ion of [Glu^1^]-fibrinopeptide B was used at 100 fmol/μL using the reference sprayer of the nanoESI source with a frequency of 30 s at 0.5 μL/min into the mass spectrometer.

### ESI-QTOF Data Processing

HDMS^E^ data were processed with Progenesis QI (Non-linear DYNAMICS, Waters) software using a Uniprot Pangasius sequence protein database (193970 entries). Propionamide as the fixed cysteine modification, oxidation as the variable methionine modification, and trypsin as the digestion enzyme were selected and one miss cleavage was allowed. Three biological and three technical replicates were used for each sample. The non-conflicting method was used as the relative quantification method. To identify statistically significant differentially expressed proteins (DEPs), the *t*-tests were adopted and proteins with at least 1.2-fold change ratio and a *p*-value < 0.05. The mass spectrometry proteomics data have been deposited to the ProteomeXchange Consortium via the PRIDE (31) partner repository with the dataset identifier PXD018364.

### Bioinformatics Analysis

The functional background annotation analyses for all the identified proteins were mapped with Gene Ontology (GO) terms^1^. The differentially expressed proteins (DEPs) were also subjected to the functional categorization of the GO Terms tool, applying the hypergeometric statistical test with correction by the Bonferroni method, considering *p*-value < 0.05 as significant. At least five proteins in each GO term focusing on immune system processes were considered as the interaction network. The enrichment analysis of statistically significant DEPs for the Kyoto Encyclopedia of Genes and Genomes (KEGG) pathway was performed using the database for annotation, visualization and integrated discovery (DAVID) Bioinformatics Resource 6.8 (32) and STRING (Search Tool for the Retrieval of Interacting Genes) software (v.11.0)^2^. At least three proteins related to the immune system in each KEGG with a requisite minimum confidence score of 0.4 were considered to be a significantly enriched pathway.

## Results

### Growth Performance

The effects of extract-based diets on the growth performance of striped catfish are shown in Table 2. At the end of the feeding trial, WG and SGR in the Pg0.2-based treatment were significantly increased compared to Pg0.08 and control diets (*p* < 0.05). The FCR values were statistically lower in fish fed Pg0.2 and Pa0.2 diets than in fish of the control treatment.

**TABLE 2:** Effects of dietary administration of single versus combination of *P. guajava* and *P. amarus* extracts on growth performance and feed utilization of *P. hypophthalmus* in W6.

**Name**	**Concentration (%)**	**WG (%)**	**SGR (%)**	**FCR**
Control	0.00	56.01 ± 11.9^a^	1.05 ± 0.18^a^	2.31 ± 0.49^a^
*P. guajava*	0.08	59.94 ± 9.83^a^	1.11 ± 0.14^a^	2.14 ± 0.38^ab^
	0.20	97.92 ± 31.5^b^	1.60 ± 0.39^b^	1.40 ± 0.53^c^
	0.50	68.20 ± 6.25^ab^	1.23 ± 0.08^ab^	1.85 ± 0.17^abc^
*P. amarus*	0.08	76.44 ± 12.2^ab^	1.34 ± 0.16^ab^	1.67 ± 0.28^abc^
	0.20	82.64 ± 14.2^ab^	1.42 ± 0.18^ab^	1.55 ± 0.29^bc^
	0.50	81.16 ± 15.4^ab^	1.40 ± 0.20^ab^	1.59 ± 0.31^abc^
Mixture	0.08	75.72 ± 21.2^ab^	1.32 ± 0.30^ab^	1.77 ± 0.59^abc^
	0.20	75.01 ± 10.8^ab^	1.32 ± 0.14^ab^	1.70 ± 0.23^abc^
	0.50	78.67 ± 7.42^ab^	1.38 ± 0.10^ab^	1.61 ± 0.16^abc^

*Values (Mean ± SEM, *n* = 3) with different superscript in the same column are significantly different (*P* < 0.05). WG, Weight gain rate; SGR, Specific growth rate; FCR, Feed conversion ratio.*

### Spleen Respiratory Burst Activity and Nitric Oxide Synthase Production

**FIGURE 1:**
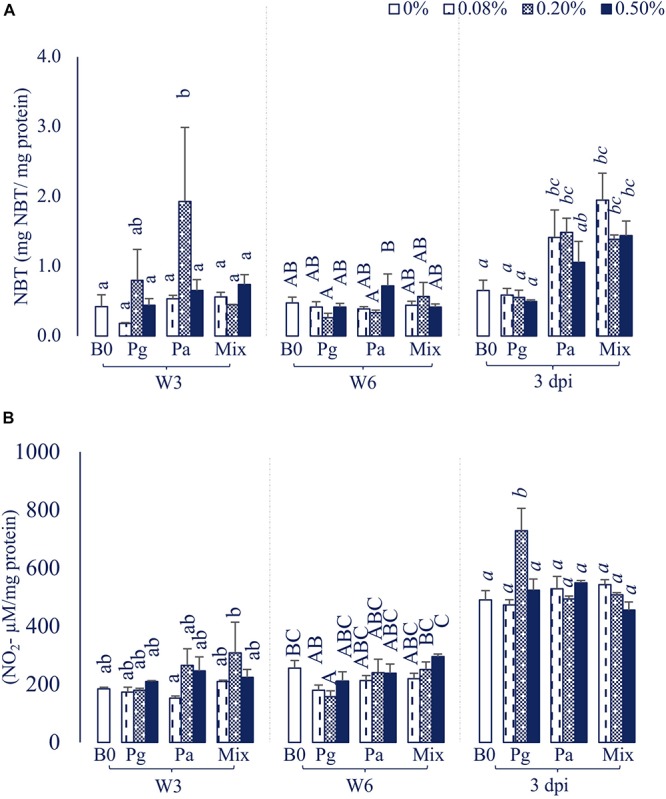
**(A)** Respiratory burst activity (RBA) and **(B)** Nitric oxide synthase (NOS) production activity in striped catfish fed extract-based diets containing a single supply or mixture of *P. guajava* and *P.* amarus at 0; 0.08; 0.2; and 0.5% after 3, 6 weeks and 3 dpi. B0, basal diet; Pg, *P. guajava*; Pa, *P. amarus;* Mix, Mixture of P. *guajava* and *P. amarus*; W3, week 3; W6, week 6; 3 dpi, 3 days post injection with *E. ictaluri*. Different letters indicate differences among diets at a given time point (*p* < 0.05). Values are means ± SEM, *n* = 3.

The Pa0.2-based diet significantly increased RBA levels compared to control in W3 (*p* < 0.05), while the levels were not statistically different between experimental and control diets in W6 (Figure 1A). However, the levels of RBA considerably increased in diets supplemented with Pa0.08, Pa0.2, and Mix (all doses) compared to Pg and control diets after injection with *E. ictaluri* (*p* < 0.05).

Single versus combination of Pg and Pa-enriched diets did not affect the NOS level compared to control in W3, although the NOS abundance in Pa0.08 was significantly lower than that of the Mix0.2 treatment (*p* < 0.05). Significantly lower levels of NOS were observed in Pg0.2-based diet compared to control in W6 (Figure 1B). The NOS activity statistically increased only in the Pg0.2-based diet compared to control after bacterial challenge (*p* < 0.05).

### Humoral and Mucosal Immune Responses

#### Lysozyme Activity

**FIGURE 2:**
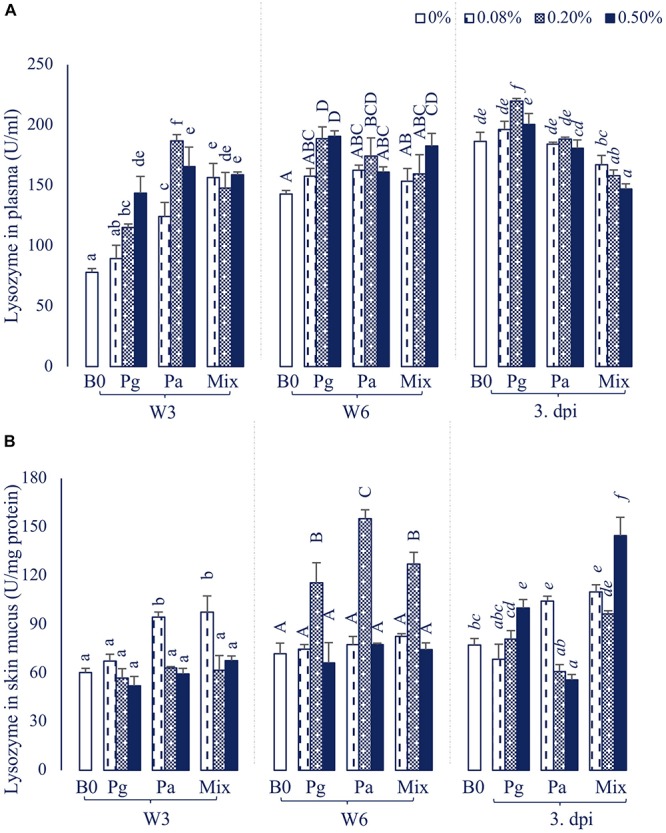
Lysozyme activity in **(A)** plasma and **(B)** skin mucus of striped catfish fed extract-based diets supplemented with a single supply or a mixture of *P. guajava* and *P. amarus* at 0; 0.08; 0.2; and 0.5% after 3, 6 weeks and 3 dpi. B0, basal diet; Pg, *P. guajava*; Pa, *P. amarus;* Mix, Mixture of P. *guajava* and *P. amarus*; W3, week 3; W6, week 6; 3 dpi, 3 days post injection with *E. ictaluri*. Different letters indicate differences among diets at a given time point (*p* < 0.05). Values are means ± SEM, *n* = 3.

The plasma lysozyme activity was significantly influenced by the extract-based diet groups (Figure 2A). In particular, the lysozyme levels were significantly increased in most of the treatments supplemented with extracts in W3, except Pg0.08 group. After 6 weeks of feeding, the plasma lysozyme activity was recorded to significantly increase in fish fed Pg, Pa0.2, and Mix0.5 (*p* < 0.05). The lysozyme level was observed to reach a maximum peak in Pg0.2 extract-based diet fed fish after intracellular injection with *E. ictaluri*, while there was a significant decrease in lysozyme levels in the three concentrations of Mix compared to control.

In parallel, the lysozyme activities in skin mucus were also significantly increased in Pa0.08 and Mix diets in W3 (Figure 2B). However, the lysozyme levels considerably increased in Pg0.2, Pa0.2 and Mix0.2 compared to control (*p* < 0.05) in W6. At 3 dpi, the lysozyme levels were significantly higher in Pg0.5, Pa0.08 and Mix (all doses) compared to control treatment (*p* < 0.05), while Pa0.5 significantly inhibited lysozyme activity compared to control.

#### Plasma Natural Hemolytic Complement Activity

**FIGURE 3:**
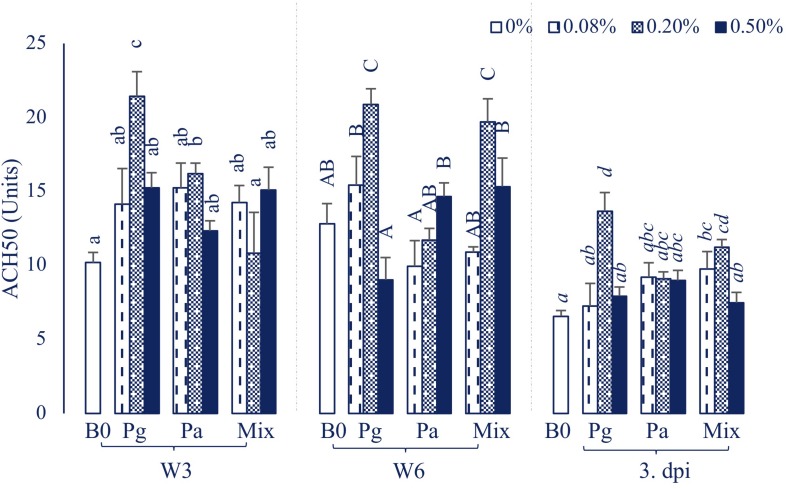
ACH50 activity in plasma of striped catfish fed extract-based diets supplemented with a single supply or a mixture of *P. guajava* and *P. amarus* at 0; 0.08; 0.2; and 0.5% after 3, 6 weeks and 3 dpi. B0, basal diet; Pg, *P. guajava*; Pa, *P. amarus;* Mix, Mixture of P. *guajava* and *P. amarus*; W3, week 3; W6, week 6; 3 dpi, 3 days post injection with *E. ictaluri*. Different letters indicate differences among diets at a given time point (*p* < 0.05). Values are means ± SEM, *n* = 3.

The treatment containing Pg0.2 was observed to possess the highest ACH50 level compared to other treatments throughout the sampling time points (Figure 3). The ACH50 activity was increased significantly in Pa0.2-based diet in W3, then the level did not statistically differ compared to control diet in W6 and 3 dpi. On the other hand, Mix0.2-based diet significantly enhanced ACH50 levels compared to control at the end of the feeding trial and post challenge with *E. ictaluri*.

#### Total Immunoglobulin

**FIGURE 4:**
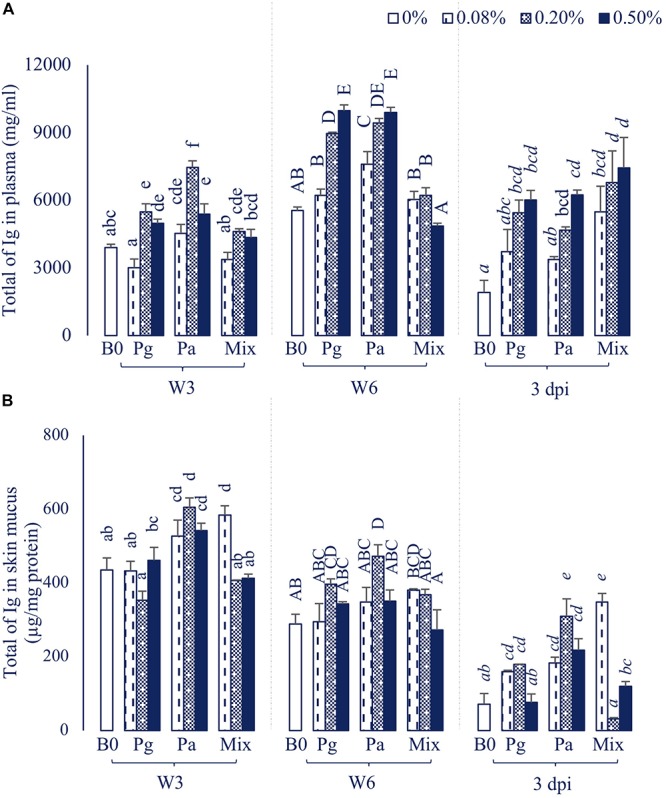
Total of Ig in **(A)** Plasma and **(B)** Skin mucus of striped catfish fed extract-based diets supplemented with a single supply or a mixture of *P. guajava* and *P. amarus* at 0; 0.08; 0.2; and 0.5% after 3, 6 weeks and 3 dpi. B0, basal diet; Pg, *P. guajava*; Pa, *P. amarus;* Mix, mixture of P. *guajava* and *P. amarus*; W3, week 3; W6, week 6; 3 dpi, 3 days post injection with *E. ictaluri*. Different letters indicate differences among diets at a given time point (*p* < 0.05). Values are means ± SEM, *n* = 3.

The statistical analysis showed that single addition of Pg or Pa extracts could strongly induce an increase of the plasma total Ig, whereas dietary administration of extract mixture did not affect the total Ig during the feeding trial (Figure 4A). Although total Ig decreased after injection with bacteria, it was significantly higher in some extract treatments than in the control group (*p* < 0.05).

Dietary administration of single or combined Pg and Pa had positive effects on total Ig in skin mucus throughout the experiment (Figure 4B). In particular, the total Ig notably increased in three concentrations of Pa-based diets in comparison to control in W3 and after the challenge test (*p* < 0.05).

#### Disease Resistance Against *E. ictaluri*

**FIGURE 5:**
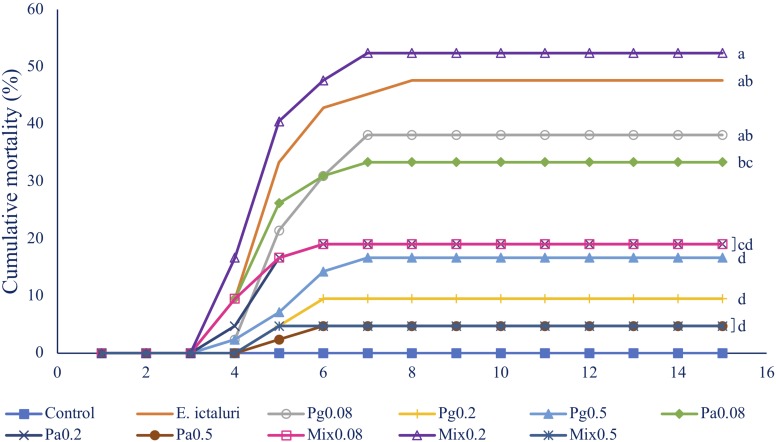
Fish mortality in 15 days after challenge test with *E. ictaluri* at week 6. B0, basal diet; Pg, *P. guajava*; Pa, *P. amarus;* Mix, mixture of P. *guajava* and *P. amarus*. Different letters indicate differences among diets (*p* < 0.05). Values are means ± SEM, *n* = 3.

Single or combined Pg and Pa extract-based diets significantly reduced striped catfish mortality after injection with *E. ictaluri* in W6 (Figure 5). The fish mortality in the control group was 47.62%. Fish fed Pa0.5 and Mix0.5 had the same lowest mortality rate (4.76%) compared to control (*p* < 0.05). Similarly, the mortalities were statistically reduced in Pg0.2, Pg0.5, Pa0.2, and Mix0.08 groups compared to control. No mortalities were observed in the negative control group. *E. ictaluri* were detected in all bacterial infection treatments.

### Effect of Extract-Based Diets on Liver Proteome Profile

#### Overall Hepatic Protein Profiling

**FIGURE 6:**
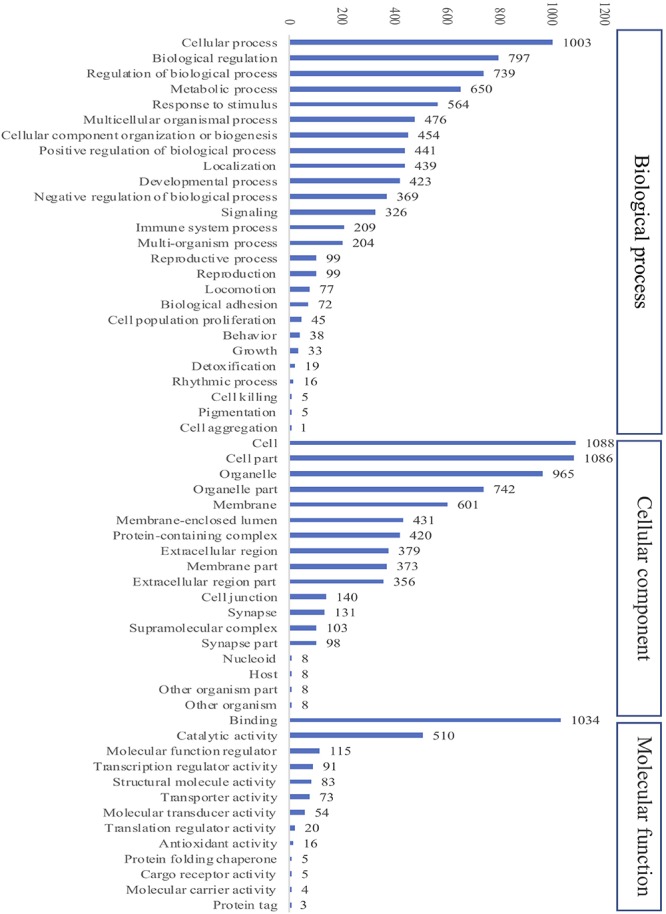
Gene Ontology (GO) analysis of all identified proteins in striped catfish liver after 6 weeks of extract-based diets. The number of proteins in three categories including Cellular component, Biological process and Molecular function.

A total of 2484 proteins were identified within the seven experimental groups of striped catfish liver with a false discovery rate of 1%, of which 1794 proteins were quantified (Supplementary Table 1). The GO annotation analysis predicted that these proteins were involved in biological process, cellular component and molecular function (Figure 6). Among the biological processes, cellular processes (55.9%) and biological regulation (44%) were the major functions of striped catfish liver. Subcategories including cells (60.6%), cell parts (60.5%), and organelles (53.8%) were commonly represented in the cellular components. Regarding molecular function, the binding subcategory was found to be the most frequent at 57.6%.

#### Comparative DEPs in Extract-Based Diets

**FIGURE 7:**
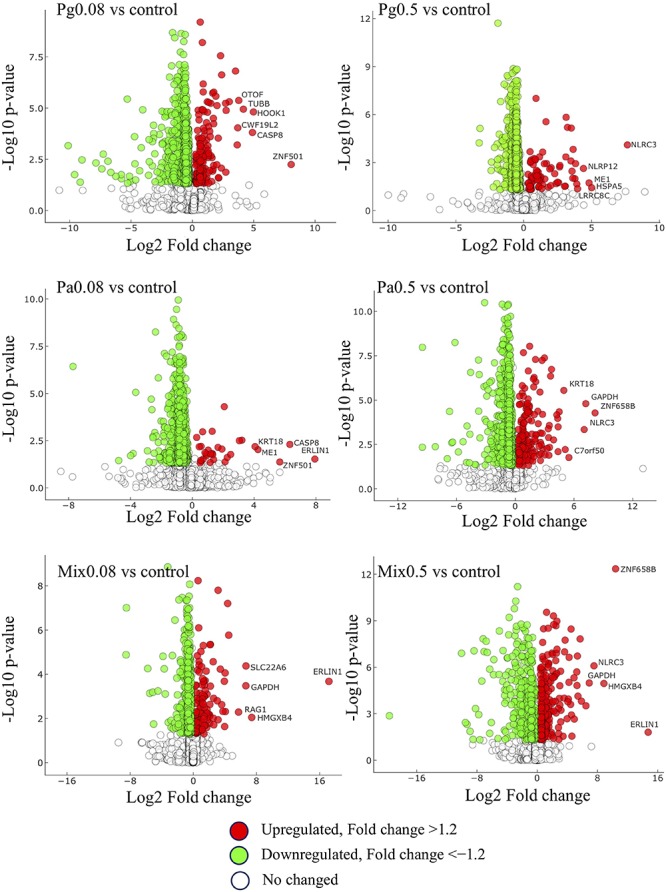
Distribution of up- and down-regulated proteins and their *P* values. Fold changes are relative to extract-based diets versus control diet. Pg, *P. guajava*; Pa, *P. amarus;* Mix, mixture of P. *guajava* and *P. amarus*. Proteins satisfying |fold change| ≥ 1.2 and *p* < 0.05. The horizontal and vertical axis represent log_2_Fold-change and −log_10_*P*-value, respectively.

The comparison of DEPs in extract-based versus control diets shown using volcano plots indicated that more proteins were downregulated than upregulated, with a fold change above 1.2 (Figure 7). Mix0.5 versus control group showed the greatest proportion (45.6%) between the number of upregulated and total DEPs compared to other extract-based groups. The lowest proportion of upregulated proteins was 3.9% in Pa0.08-based diet versus control, followed by Pg0.5 versus control (9.9%), Pg0.08 versus control (19%), Mix0.08 versus control (22.2%), and Pa0.5 versus control (22.6%). In contrast, the number of downregulated DEPs in each extract-based diet versus control was the highest in Pa0.08 and the lowest in Mix0.5. Moreover, glyceraldehyde-3-phosphate dehydrogenase (GAPDH), NLR family CARD domain-containing protein 3 (NLRC3), caspase-8 (CASP8), zinc finger protein 501 (ZNF501), Erlin-1 (ERLIN1), keratin-type I cytoskeletal 18 (KRT18), and HMG domain-containing protein 4 (HMGXB4) were always represented as the top five upregulated proteins in at least two extract-based treatments.

### Extract-Based Diets Affect the Liver Proteome Network Related to Antioxidant, Immune, and Stress Responses

#### GO Functional Enrichment Classification of the DEPs

**FIGURE 8:**
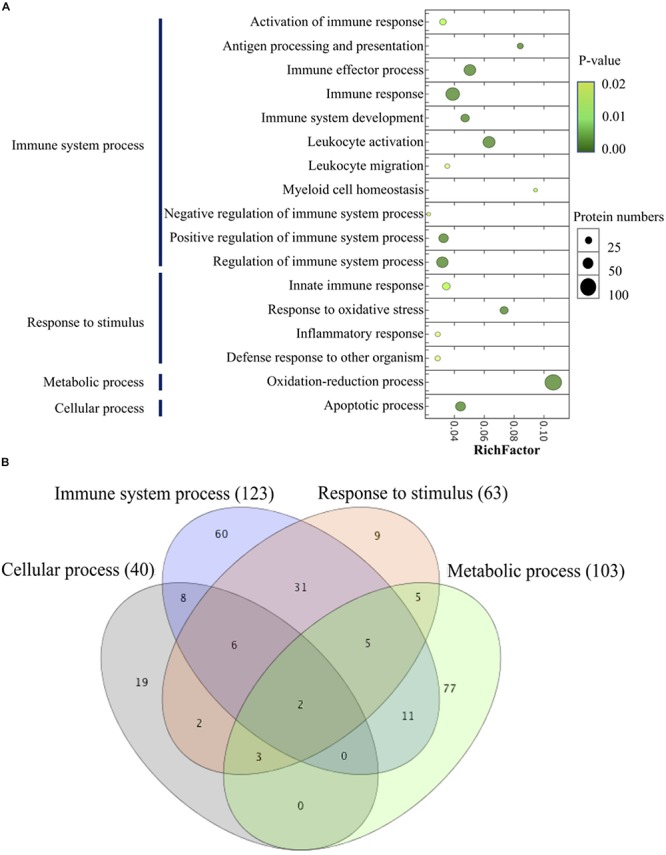
Scatter plot of GO enrichment analyses **(A)** and hierarchical clustering **(B)** of differentially abundant proteins related to immune system process. The *Y*-axis is different GO functions. The *X*-axis is the value of rich factor (*p*-value of Fisher exact test < 0.05). The color of circles stands for the *p*-value of function. The size of circles stands for protein numbers.

By performing the GO enrichment analysis, 238 DEPs focusing on immune system processes, response to stimulus (innate immune response, response to oxidative stress, inflammatory response, and defense response to other organisms), a metabolic process (oxidation-reduction process) and cellular process (apoptotic process) were classified. The results indicated that there were 11 GO clusters belonging to immune system processes, with the most abundant DEPs grouped into immune response (71 proteins), followed by leukocyte activation (57 proteins) and immune effector process (54 proteins), whereas leukocyte migration and myeloid cell homeostasis (10 proteins) were less abundant in this group. Up to 103 proteins were involved in the oxidation-reduction process of metabolism; 63 proteins were classified into the subclasses of response to stimulus; and 40 proteins related to the apoptotic process (Figure 8A and Supplementary Table 2). The comparison of the different GO annotations indicates that there was a strong overlap between the GO functions (Figure 8B). Two DEPs including PRDX3 (peroxiredoxin-3) and GAPDH were represented in all the four GO enriched pathways.

**FIGURE 9:**
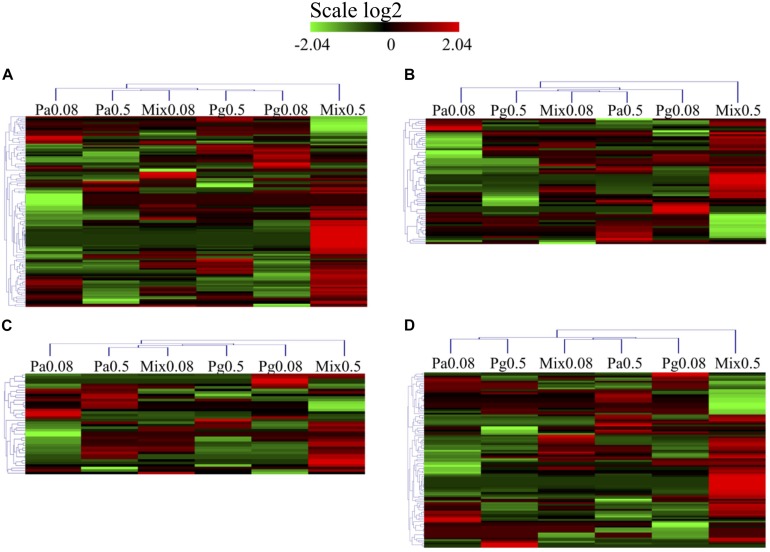
Heatmap of GO and protein domain enrichment analysis of DEPs related to **(A)** immune system process. **(B)** Response to stimulus (response to stress). **(C)** Apoptotic process. **(D)** Oxidation-reduction process in extract-based diets compared to control diet. Heatmap represents differentially expressed proteins in extract-enriched diets (|fold change| > 1.2, *p* < 0.05).

Regarding the functional categories mentioned above, 238 DEPs were measured across the groups of extract-based diets with the control treatment (Figure 9 and Supplementary Table 3). Mix0.5 group induced upregulation in all GO categories, the ratios between upregulated versus downregulated DEPs being 52 versus 19 (immune system process), 24 versus 15 (response to stimulus), 17 versus 6 (apoptotic process), and 36 versus 33 (oxidation-reduction process). GO enrichment analysis also revealed that the apoptotic process and response to stimulus categories were upregulated in the Pa0.5-based diet, while the Mix0.08 group could induce the upregulation of apoptotic process. Enriched proteins were mostly downregulated in all tested categories in Pg0.08, Pg0.5, and Pa0.08 groups. Among the GO enriched categories, six proteins coding for the NLR family CARD domain-containing protein 3 (NLRC3), tubulin beta chain (TUBB), glyceraldehyde-3-phosphate dehydrogenase (GAPDH), NACHT, LRR and PYD domains-containing protein 12 (NLRP12), recombination activating protein 1 (RAG1), and caspase-8 (CASP8) were highly upregulated in immune system processes. In response to stimulus, proteins namely CASP8, GAPDH, and keratin (KRT18) were highly upregulated. The expressions of two proteins encoding GAPDH, and TUBB were highly increased in the apoptotic process. Only the GAPDH protein was highly expressed in the oxidation-reduction process.

#### Differentially Regulated KEGG Pathways Related to Antioxidant and Immune Response Among Extract-Based Diets

**FIGURE 10:**
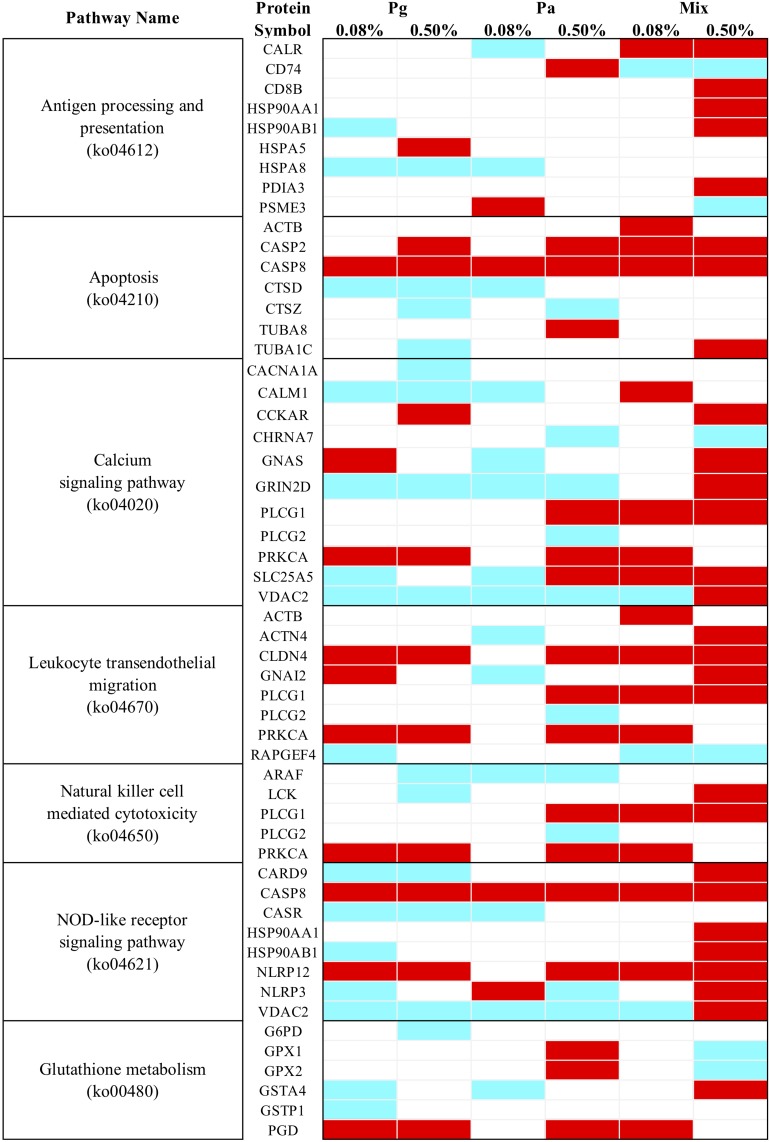
Heatmap of KEGG pathway enrichment analysis for immune and antioxidant related DEPs. Cyan, significant downregulation, Red, significant upregulation, White, no change.

All the DEPs were mapped in the KEGG database to search for the proteins involved in significant immune and antioxidant-related pathways. The results indicated that in total seven immune and antioxidant related KEGG pathways were significantly enriched in most extract-based treatments (*p* < 0.05) (Figure 10). In comparison to the control diet, the Mix0.5-based diet significantly upregulated the expression of proteins that were involved in antigen processing and presentation, apoptosis, leukocyte transendothelial migration, natural killer cell mediated cytotoxicity, and some signaling pathways such as calcium and NOD-like receptor. Moreover, upregulated proteins involved in pathways related to apoptosis, leukocyte transendothelial migration, natural killer cell mediated cytotoxicity, and the calcium signaling pathway were found in Mix0.08. Regarding Pa extract-based diets, Pa0.5 induced the upregulation of proteins involved in some KEGG pathways including apoptosis, leukocyte transendothelial migration, glutathione metabolism and oxidative phosphorylation. In parallel, most of the proteins related to the calcium signaling pathway and leukocyte transendothelial migration decreased in expression following Pa0.08-based diet. On the other hand, in Pg0.5-based diet leukocyte transendothelial migration related proteins were upregulated, whereas proteins related to calcium signaling pathway were downregulated. Upregulated proteins involved in leukocyte transendothelial migration as well as downregulated proteins in antigen processing and presentation, calcium and NOD-like receptor signaling pathways were also enriched in the Pg0.08 group.

Among the nine proteins predicted to be related to antigen processing and presentation, T-cell surface glycoprotein CD8 beta chain (CD8B), heat shock protein HSP 90-alpha 1 (HSP90AA1), HSP 90-beta (HSP90AB1), and protein disulfide-isomerase A3 (PDIA3) additively increased in the Mix0.5 group compared to other experimental groups. CASP8 predicted to belong to both NOD-like receptor signaling and apoptosis pathways, significantly increased in all extract treatments. Several proteins such as tubulin alpha-1C chain (TUBA1C), cholecystokinin receptor type A (CCKAR), guanine nucleotide-binding protein G(s) subunit alpha (GNAS), glutamate receptor ionotropic NMDA 2D (GRIN2D), PLCG1 (1-phosphatidylinositol 4,5-bisphosphate phosphodiesterase gamma-1), PRKCA (protein kinase C alpha type), ADP/ATP translocase 2 (SLC25A5), voltage-dependent anion-selective channel protein 2 (VDAC2), alpha-actinin-4 (ACTN4), guanine nucleotide-binding protein G(i) subunit alpha-2 (GNAI2), tyrosine-protein kinase Lck (LCK), caspase recruitment domain-containing protein 9 (CARD9), NACHT, LRR, and PYD domains-containing protein 12 (NLRP12) and NLRP3 were differentially expressed depending on the dose of extracts. Furthermore, most DEPs involving glutathione metabolism [i.e., glutathione peroxidase 1 (GPx1), glutathione peroxidase 2 (GPx2), and 6-phosphogluconate dehydrogenase (decarboxylating) (GPD)] were upregulated in Pa0.5 group only.

#### DEPs Involved in Lipid Metabolism

Dietary supplementation with Pg and Pa extracts significantly altered protein expression levels related to lipid metabolism in plant extract-based groups compared to the control group. KEGG pathway enrichment analysis showed that a total of 15 DEPs were reported to be distributed into four significant subcategories involved in lipid metabolism such as fatty acid degradation [fatty acid metabolism, glycerolipid metabolism, and sphingolipid signaling pathways (Table 3)]. Most proteins were significantly downregulated in at least one extract group compared to the control group. However, only protein kinase C alpha type (PRKCA) belonging to the sphingolipid signaling pathway was highly upregulated in four groups including Pg0.08, Pg0.5, Pa0.5, and Mix0.08; guanine nucleotide-binding protein G(i) subunit alpha-2 (GNAI2) of the sphingolipid signaling pathway and 4-trimethylaminobutyraldehyde dehydrogenase (ALDH9A1) related to glycerolipid metabolism were upregulated in Mix0.5 group only; long-chain specific acyl-CoA dehydrogenase (mitochondrial ACADL) related to fatty acid degradation and fatty acid metabolism significantly increased in the Pa0.5 group; alcohol dehydrogenase 5 class-3 (ADH5) related to fatty acid degradation considerably increased in expression in the Pa0.08 group.

**TABLE 3:** List of differentially express proteins in striped catfish liver related to lipid metabolism after dietary administration plant extracts.

**Description**	**Accession**	**Protein name**	**Pg**		**Pa**		**Mix**		**Mass (×10^3^)**
			0.08	0.5	0.08	0.5	0.08	0.5	
**Fatty acid degradation (KEGG:ko00071)**									
Long-chain specific acyl-CoA dehydrogenase- mitochondrial	AHH41666.1	ACADL				1.3			49.4
Peroxisomal acyl-coenzyme A oxidase 1	XP_017337623.1	ACOX1	−1.2						74.7
Peroxisomal acyl-coenzyme A oxidase 3	XP_017327584.1	ACOX3						−2.2	78.9
Alcohol dehydrogenase class-3	XP_017548877.1	ADH5	−1.6		2.0			−1.3	41.8
Aldehyde dehydrogenase- mitochondrial	XP_017307563.1	ALDH2	−1.6	−1.6	−1.3	−1.9	−1.5	−1.4	57.4
Fatty aldehyde dehydrogenase	XP_022531853.1	ALDH3A2	−1.7			−1.6			55.1
Alpha-aminoadipic semialdehyde dehydrogenase	XP_007252494.1	ALDH7A1	−2.0	−1.9		−2.1	−1.4	−1.4	59.3
4-trimethylaminobutyraldehyde dehydrogenase	XP_007250712.2	ALDH9A1			−1.9			1.2	57.0
Trifunctional enzyme subunit beta- mitochondrial	XP_007260524.2	HADHB	−1.6	−1.6	−1.4	−1.7	−1.6	−1.3	50.6
**Fatty acid metabolism (KEGG:ko01212)**									
Long-chain specific acyl-CoA dehydrogenase- mitochondrial	AHH41666.1	ACADL				1.3			49.4
Peroxisomal acyl-coenzyme A oxidase 1	XP_017337623.1	ACOX1	−1.2				−1.2		74.7
Peroxisomal acyl-coenzyme A oxidase 3	XP_017327584.1	ACOX3						−2.2	78.9
Trifunctional enzyme subunit beta- mitochondrial	XP_007260524.2	HADHB	−1.6	−1.6	−1.4	−1.7	−1.6	−1.3	50.6
**Glycerolipid metabolism (KEGG:ko00561)**									
1-acyl-sn-glycerol-3-phosphate acyltransferase epsilon	AHH40462.1	AGPAT5			−1.9				42.4
Aldose reductase	XP_017321050.1	AKR1B1	−1.7	−2.0	−1.4	−1.8	−1.3	−1.5	36.8
Aldehyde dehydrogenase mitochondrial	XP_017307563.1	ALDH2	−1.6	−1.6	−1.3	−1.9	−1.5	−1.4	57.4
Fatty aldehyde dehydrogenase	XP_022531853.1	ALDH3A2	−1.7			−1.6			55.1
Alpha-aminoadipic semialdehyde dehydrogenase	XP_007252494.1	ALDH7A1	−2.0	−1.9		−2.1	−1.4	−1.4	59.3
4-trimethylaminobutyraldehyde dehydrogenase	XP_007250712.2	ALDH9A1			−1.9			1.2	57.0
**Sphingolipid signaling pathway (KEGG:ko04071)**									
Cathepsin D	XP_007250635.1	CTSD	−1.2	−1.5	−1.3				43.6
Guanine nucleotide-binding protein subunit alpha-13	XP_007259155.2	GNA13						−33.6	44.3
Guanine nucleotide-binding protein G(i) subunit alpha-2	XP_022531259.1	GNAI2			−1.7			1.4	40.9
Protein kinase C alpha type	XP_017314924.1	PRKCA	4.3	2.0		3.2	2.6		79.5

## Discussion

Manipulation of health status using plant extracts has been developed as an ecological practice for sustainable aquaculture (3, 33). Among the plant derived products, Pg and Pa displayed antioxidant and immunomodulatory properties due to their presence of various biological compounds (6, 34–37). In the present study, the immunomodulatory effects of Pg and Pa extract alone or in combination were assessed by evaluating their capacities to improve immune parameters and resistance to *E. ictaluri* infection. Notably, this study also provided an insight into the changes observed in the immune proteome of striped catfish liver after feeding with extract-based diets.

### Growth Performance and Feed Utilization

After 6-weeks of feeding, the highest values of WG and SGR were observed in Pg0.2 groups, confirming that Pg supplemented at its optimal dose could improve the growth of striped catfish. FCR values lower than control were observed in most of the plant extract-based treatments, but the significantly lower FCR values were only achieved in the groups fed Pg0.2 and Pa0.2 diets. The lower FCR value in fish fed plant extract-based diets suggests an improvement in feed utilization for fish growth, thus reducing the cost of production. However, the enhancement of growth performance by extract-based diets may occur in a fish species, time and concentration-dependent manner. Giri et al. (8) indicated that guava leaf-enriched diets at the high concentration (0.5%) significantly increased the growth performances of rohu in 60 days. The growth and the nutritional indicators of Nile tilapia *Oreochromis niloticus* fingerlings were considerably improved after 84 days of feeding with 0.5, 0.75, and 1% ethanol guava extracts (38). Another study by Gobi et al. (10) reported that a considerable increase of final weight and SGR of *O. mossambicus* as well as a decrease of FCR were observed with a 1% ethanol guava leaf extract-based diet in a shorter time of 30 days of feeding.

### Immune Response

#### Cellular Immune Response

Phagocytes constitute the primary limitation of invasive pathogens by releasing ROS and NOS (39, 40). Our study highlighted that the spleen RBA significantly increased in only Pa0.2-based diet compared to control in W3, whereas the NOS level was found to significantly decrease in Pg0.2-based diet at the same time. The results also could not detect significant differences in RBA and NOS activities in all extract-based diets compared to control in W6. In line with our results, the Pg derived products could stimulate the increase of NOS production in several aquatic animal species including giant tiger prawn (*Penaeus monodon*) (41), koi carp (*Cyprinus carpio* var. *koi* L.) (42), and tilapia (10), although there are a limited number of scientific papers regarding the effects of Pa extract on the NOS activity of aquatic species. However, our further study demonstrated that Pa ethanol extract and its fractions (dichloromethane and ethyl acetate) significantly inhibited the NOS production, whereas Pg ethanol extract and its fractions enhanced the production of NOS in striped catfish head kidney leukocytes (data not shown). Moreover, significant dose-dependent inhibitions of NOS production were observed in male Balb/c mice after 14 days of Pa administered by oral gavage (34). It could be explained that the NOS level increased in the single diet supplied with 0.2% Pg extract but the level did not increase in mixture diets after challenge test. Regarding RBA activity, tilapia fed diets supplemented with 0.1, 0.5 and 1.0% ethanol guava extract displayed significantly higher RBA levels at day 30 (*p* < 0.05) (10). Similarly, the leaf of *P. guajava* and mango *Mangifera indica* extract-based diets also significantly enhanced RBA production in rohu after a 35-day feeding period (9). As mentioned above, ROS are generated by neutrophils and macrophages during bacterial infection, while NOS is created as a signaling molecule in ROS generation during phagocytosis (43). Enhanced activities of ROS and NOS are seen as indicators of immune condition. Our study also found that the RBA and NOS levels were highly increased in all treatments after *E. ictaluri* infection. Notably, the RBA levels were statistically enhanced in Pa0.08, Pa0.2, and Mix (all dose) treatments compared to control, whereas only the Pg0.2-enriched diet could stimulate the increase of NOS after the challenge test (*p* < 0.05).

#### Humoral Immune Response

In aquatic animals, the humoral innate immune system has been considered as a vital weapon in protecting fish against opportunistic pathogens (44). The current study revealed that the increase of serum lysozyme, complement activities, as well as total immunoglobulin levels was concentration and time-dependent in extract-based diets. In agreement with our results, several studies also indicated that Pg or Pa extract-based diets positively enhanced lysozyme, complement activities and total Ig in rohu (8, 9), tilapia (10), common carp (11), and striped catfish (14). After infection by *E. ictaluri*, the lysozyme and complement activities as well as the total Ig mostly decreased but were still significantly higher in some extract-based diets compared to those of the control. These results are consistent with our previous study showing that the humoral immune parameters (i.e., lysozyme, complement activities, and total Ig) in Pg and Pa-based diets were significantly higher than those of the control diet after a bacterial challenge test (14).

Skin is a major line of entry for infectious pathogens in aquatic animals which contains various biologically active components (including defensive molecules) (45). In the present study, single or combined administrations of Pg and Pa differentially enhanced the lysozyme and total Ig in skin mucus of striped catfish. In line with these results, we previously also found that Pg and Pa extract-based diets at 0.2 and 1.0% considerably enhanced lysozyme activity as well as total Ig in skin mucus after an 8-week feeding period (14). Moreover, the skin mucus total Ig was also considerably increased in common carp fed 1.0% Pg leaf extract-based diet at week 8 (11).

The elevation of the cellular immune response in association with humoral immune response in serum and skin mucus contributes to enhance the defense mechanism by destroying invading pathogens. Our findings also revealed that single or combined Pg and Pa extract-based diets had a positive influence on the survival of striped catfish after *E. ictaluri* infection, especially in Pg0.2, Pg0.5, Pa0.2, Pa0.5, Mix0.08, and Mix0.5 groups, and the combination of Pg and Pa extracts had a positive impact on the disease resistance of striped catfish. Improving the survival rates in striped catfish against *E. ictaluri* infection demonstrated in this study were in agreement with the previous reports that indicated Pg extract-based diets significantly reduced mortalities in rohu (8, 9), tilapia (10), and Nile tilapia (38) after challenge with *A. hydrophila*.

#### Liver Proteome Profile

The liver is one of the most important organs that participates in metabolism and nutrient storage, as well as the immune response (18). The current findings revealed that cellular processes and biological regulation were the major biological processes affected in striped catfish liver. The top three subcategories including cells, cell parts and organelles were the majority in cellular components. Regarding molecular functions, the subcategory of bindings was found to be most abundant. Similar contributions of the subcategories were also suggested by Wen et al. (20), although the metabolic process was found to be the second major biological process in obscure pufferfish liver. However, cellular processes and metabolic processes were the top two subcategories of biological processes in mice liver treated with sucralose (46). Our results also indicated that a number of proteins were differentially expressed according to the dose and species of plant extracts added to striped catfish diets. When compared to control, Mix0.5 showed the highest proportion of upregulated proteins and, more globally, total DEPs. These proportions were reduced in Pa0.5, Mix0.08, Pg0.08, Pg0.5, and Pa0.08. This outcome suggests that both single and combined Pg and Pa extract-based diets directly changed the expression of proteins in striped catfish liver.

#### Effect of Extract-Based Diets on Regulating Immune System Functions and Antioxidant Metabolism

It was reported that antigen processing and presentation play a key role in adaptive immunity in teleost fish (47, 48). The antigen processing and presentation pathway was also enriched in beef cattle fed a mixture containing dry corn grain, corn silage, soybean, citrus pulp pellets, urea, calcareous, mineral salt, and potassium chloride (49). Our results showed that most of the proteins involved in antigen processing and presentation were significantly upregulated in Mix-based diets (Mix0.08 and Mix0.5), while those protein expressions were inhibited in Pg0.08. Among nine proteins belonging to antigen processing and presentation, CD8B was significantly upregulated in Mix0.5 group, although the expression level of CD8B was not changed in single diets supplemented only by Pg or Pa. CD4^+^ and CD8^+^ are vital in modulating the immune response in the regulation of cytokines (50). In Wistar-Kyoto rats, Pa possessed effective immunosuppressive activities in the cellular immune response. There was a significant decrease in the expression of *cd4*+ and *cd*8+ genes in spleen and serum of rats after treatment with Pa in the presence of lipopolysaccharide (34). Phyllanthin isolated in Pa also caused a significant reduction in the percentage expression of *cd4*+ and *cd*8+ genes when supplemented in Balb/C mice diets (51). In the present study, HSP90AA1 and HSP90AB1 were predicted to be involved in both antigen processing and presentation and NOD-like receptor signaling pathways. Moreover, both HSP90AA1 and HSP90AB1 expressions were significantly upregulated in the Mix0.5 group, whereas HSP90AB1 level was reduced in the Pg0.08 group only. Upregulation of *hsp90* following curcumin-based diets in pool barb *Puntius sophore* has also been reported by Mahanty et al. (52). A previous study suggested that in loach *Misgurnus anguillicaudatus* HSP70, HSP90 alpha and HSP90 beta, which are associated more specifically with thermal stress, were strongly upregulated after the fish were fed vitamin C (53). Cold stress-low temperature was also proven to induce HSP90 transcriptional expression in pufferfish liver (54). PDIA3 is mainly present in the endoplasmic reticulum and is crucially involved in the folding process of MHC class I (55). CALR-calreticulin is also important in MHC class I antigen processing and presentation. The upregulation in expression of CD8B, PDIA3, CALR as well as HSP90AA1, and HSP90AB1 in the liver could be associated with the activation of the antigen processing and presentation pathway in the Mix0.5 group.

The leukocyte transendothelial migration pathway has also been associated with adhesion molecules, chemokines and cytoskeletal regulators (56). In response to inflammatory signals, leukocytes leave the bloodstream by crossing the endothelial monolayer which results in changes to the adhesive properties and shape of cells (56). Moreover, several molecules in the leukocyte transendothelial migration pathway were also activated in response to immune challenge at different metamorphosis stages in the grouper (*Epinephelus coioides*) (57). In particular, several proteins present in the leukocyte transendothelial migration pathway were significantly upregulated in striped catfish fed Pg0.08, Pg0.5, Pa0.5, Mix0.08, and Mix0.5.

Moreover, natural killer cell mediated cytotoxicity is essential for the first line immune defense against invading pathogens as well as for modulation of liver injury (58). Several proteins involved in natural killer cell mediated cytotoxicity were also noticeably upregulated in Mix groups. Calcium signaling plays a critical role in many cellular processes including activation of inflammasomes (59). Increasing intracellular calcium could damage the mitochondria and then activate the NLRP3 inflammasome through the release of ROS (60). The present results also revealed increased expression of three proteins (PRKCA, PLCG-1 and 2) related to natural killer cell mediated cytotoxicity, the calcium signaling pathway and leukocyte transendothelial migration, suggesting a link between these immune subcategories.

The NOD-like receptor signaling pathway contributes to many crucial activities including autophagy, apoptosis, and development (61). In humans, NOD-like receptors act as major players in the interface between innate immunity and cancer. The NOD-like receptor family of proteins is vital in mediating the initial innate immune response, as well as having roles in cellular injury and stress (62). Of the 8 proteins involved in the NOD-like receptor signaling pathway, seven proteins were highly upregulated with the Mix0.5-based diet. NLRP3 is an intracellular sensor whose activation leads to the formation of an inflammasome in leukocytes (62). Specifically, NLRP3 positively regulates apoptosis through the activation of CASP8 (63). Similarly, NLRP12 acts as a proinflammatory protein in caspase-1 signaling (64). NLRP12 was upregulated in most of the plant extract groups, except in Pa0.08, whereas extract-based diets dysregulated the expression of NLRP3 in a dose-dependent manner.

The present study also found that many proteins involved in apoptosis were significantly upregulated in Pa0.5, Mix0.08, and Mix0.5 groups. Apoptosis is a physiological program that is a type of programmed cell death and plays a key role in the regulation and functioning of the immune system (65). In the liver, apoptosis is a physiological process to eliminate damaged or infected cells and maintain tissue homeostasis (66). The apoptosis process is mediated by a family of aspartate-specific cysteine proteases known as caspases (67). Notably, our results revealed the level of CASP8 considerably increased in all extract-based diets compared to control, which was predicted to be involved in both the NOD-like receptor signaling pathway and apoptosis. In parallel, the expression of CASP2 was statistically increased in Pg0.5, Pa0.5, Mix0.08, and Mix0.5. In line with our results, Van et al. (68) also demonstrated that guava leaf extract increased the expression of proteins involved in apoptotic processes including caspases 3, 8 and 9 in HepG2 cells. The same results were reported by Mbaveng et al. (2016), indicating that Pg bark extract induced apoptosis in leukemia CCRF-CEM cells via caspase (caspase 3/7, 8, and 9) activation (69). Similarly, Pa induced apoptosis in Dalton’s lymphoma ascite cells in mice through activation of *casp3* mRNA (70). These effects might be due to the presence of phenolic compounds in the extract (71, 72). The activation of caspases results in DNA fragmentation, destruction of the nuclear proteins and cytoskeleton, crosslinking of proteins, expression of ligands for phagocytic cells, and formation of apoptotic bodies (73, 74). It should be noted that the combination of Pg and Pa extracts resulted in the upregulation of higher levels of proteins related to antigen processing and presentation, natural killer cell mediated cytotoxicity, the calcium signaling pathway and the NOD-like receptor signaling pathway, suggesting possible synergistic effects of these bioactive compounds when compared to plant extracts alone.

Furthermore, the proteomic analysis in the liver also revealed different regulations of glutathione metabolism among experimental groups compared to the control group. Notably, six proteins were directly regulated and functionally related to antioxidation. The results suggested that the Pa0.5-based diet had significant effects on the regulation of antioxidation in striped catfish liver. It has been shown that the presence of phenolic compounds in plant extracts greatly contributes to their antioxidant potential (75, 76). Furthermore, Pa contains a rich source of hydrolyzable phenols including phyllanthin and hypophyllanthin (77) which are highly responsible for antioxidant activity (78). Among the proteins, glutathione peroxidase is important in preventing many molecules from being damaged by oxidative effects during oxidative stress (79, 80). Antioxidant enzymes including superoxide dismutase (SOD), catalase (CAT) and glutathione peroxidase (GPx) were also increased in tilapia fed guava leaf extract (10), whereas Pa significantly stimulated the increase of GPx activity in rat plasma (81). In our study, glutathione peroxidase 1 (GPx1) and glutathione peroxidase 2 (GPx2) expression were upregulated in the Pa0.5 group, while both proteins were downregulated in the Mix0.5 group. These findings suggest that the combination of Pa and Pg did not support any possible synergistic effect on antioxidant activity.

#### Effect of Extract-Based Diets on Lipid Metabolism

Single or combination Pg and Pa extract-based diets significantly reduced protein abundances related to lipid metabolism (i.e., fatty acid degradation, fatty acid metabolism, glycerolipid metabolism, and sphingolipid signaling pathways), suggesting a negative effect of the extracts on the lipid metabolism process in striped catfish liver. This could explain why growth did not significantly increase in 0.08 and 0.5% extract-based diets in W6. Lipid metabolism is a complex process and the key proteins related to this process play important roles in animals (82). Our results showed that only PRKCA, which belongs to the sphingolipid signaling pathway, was considerably upregulated in Pg0.08, Pg0.5, Pa0.5, and Mix0.08 groups. Long-chain specific acyl-CoA dehydrogenase (ACADL) is thought to be involved in both fatty acid degradation and fatty acid metabolism and was upregulated in the Pa0.5 group only. The decrease of protein levels related to fatty acid synthase was observed in rainbow trout liver following handling stress (83). However, dietary fish oil mixture (EPA/DHA 1:1) consumed together by rats could upregulate fatty acid beta oxidation (ACOX2) after 24 weeks of feeding (19).

## Conclusion

**FIGURE 11:**
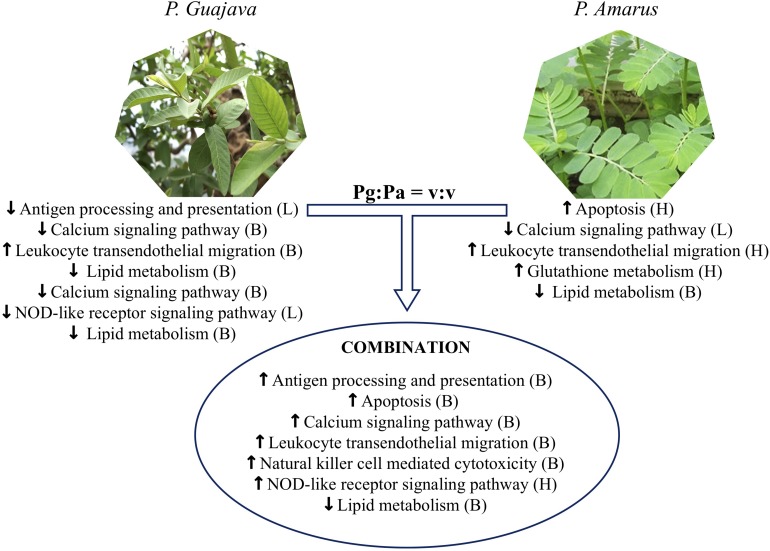
Metabolic pathways in striped catfish liver altered by single versus combination Pg and Pa extract-enriched diets as compared to control diet. L, Low dose examined (0.08%), H, high dose examined (0.5%) and B, both high and low doses.

In conclusion, our findings revealed how single and combined Pg and Pa extract-based diets acted in the regulation of immune responses, significantly reducing fish mortality. They also provide a better understanding about the effects of these plant extracts, alone or in combination, on the liver proteome of striped catfish. The results of humoral and cellular immune responses throughout the feeding experiment, as well as the reduced mortality recorded after the bacterial challenge test, supported additive effects of the plant extract mixture compared to the single extracts at the dose of 0.08%. The proteomic results showed that the administration of combined *P. guajava* and *P. amarus* extracts could modulate proteins involved in antigen processing and presentation, leukocyte transendothelial migration, natural killer cell mediated cytotoxicity, the calcium signaling pathway, the NOD-like receptor signaling pathway and apoptosis in striped catfish, whereas single Pg or Pa-enriched diets stimulated the upregulation of fewer pathways (Figure 11). Only the diet supplemented with 0.5% *P. amarus* could enhance the upregulation of glutathione metabolism. In addition, all extract treatments induced the downregulation of pathways related to lipid metabolism. Moreover, the proteomics data also showed that diets supplemented with mixture of *P. guajava* and *P. amarus* resulted in the additive increased expression of several proteins such as CD8B, HSP90AA1, HSP90AB1, PDIA3, CASP2, CASP8, TUBA1C, CCKAR, GNAS, GRIN2D, PLCG1, PRKCA, SLC25A5, VDAC2, ACTN4, GNAI2, LCK, CARD9, NLRP12, and NLRP3. This outcome suggests a potential synergistic effect of combining both plant extracts for the regulation of striped catfish immune responses, but not on antioxidant activity. The main constituents of the extracts should be identified and quantified, as this can influence the modulation of immune capacity and health in fish.

## Data Availability Statement

The mass spectrometry proteomics data have been deposited to the ProteomeXchange Consortium with the dataset identifier PXD018364. Other raw data supporting the conclusions of this article will be made available by the authors, without undue reservation, to any qualified researcher.

## Ethics Statement

Ethical review and approval was not required for the animal study because we performed the experiment in Vietnam. Our country has not yet established the animal ethics committee. However, after we sampled the fish, we anesthetized them by immersion in ethyl-aminobenzoic acid. Then fish were euthanized by cervical dislocation.

## Author Contributions

PK, JQ-L, M-LS, NP, BH, and DH conceived and designed the project. LB prepared the plant extracts. MO, ND, and TN performed the experiment. TN analyzed and interpreted the data, wrote, and revised the manuscript. VC interpreted the proteomic data. PK, VC, and BH revised the manuscript. All the authors approved the manuscript.

## Conflict of Interest

The authors declare that the research was conducted in the absence of any commercial or financial relationships that could be construed as a potential conflict of interest.

## Footnotes

1 http://geneontology.org/

2 http://string-db.org
